# 

**Published:** 1895-03

**Authors:** 


### TABLE OF CONTENTS.

### ORIGINAL COMMUNICATIONS.

PAGE

Moderation in Practice and in Statement. By SAFFORD G. PERRY, D.D.S., New York City . . . . . . . . . . . . . . . . . . . . . . . . . . . . 133

Cancer of the Tongue. By E. W. STEVENS, M.D., Philadelphia . . . . . 146

The Relation of Modern Therapeutics to the Practice of Dentistry. By ROBERT W. GREENLEAF, M.D., Boston, Mass. . . . . . . . . . . . . . 152

### REPORTS OF SOCIETY MEETINGS.

New York Odontological Society . . . . . . . . . . . . . . . . . . . . . 160

Academy of Stomatology . . . . . . . . . . . . . . . . . . . . . . . . . 165

### EDITORIAL.

The Loyalty of Society Members . . . . . . . . . . . . . . . . . . . . . 182

Entrance Examinations to Dental Colleges . . . . . . . . . . . . . . . . 184

### BIBLIOGRAPHY . . . . . . . . . . . . . . . . . . . . . . . . . . . . 186

### CURRENT NEWS.

Annual Meeting of the International Dental Publication Company . . . . 192

The Horace Wells Permanent Memorial, under the Auspices of the American Dental Association . . . . . . . . . . . . . . . . . . . . . . . . . 194

First District Dental Society of New York . . . . . . . . . . . . . . . 195

### NOTES AND COMMENTS . . . . . . . . . . . . . . . . . . . . . . . . . 196

